# How Physical Proximity Shapes Complex Social Networks

**DOI:** 10.1038/s41598-018-36116-6

**Published:** 2018-12-07

**Authors:** Arkadiusz Stopczynski, Alex ‘Sandy’ Pentland, Sune Lehmann

**Affiliations:** 1 Technical University of Denmark, Lyngby, Denmark; 2 Media Lab, Massachusetts Institute of Technology, Cambridge, MA USA; 3 Niels Bohr Institute, University of Copenhagen, Copenhagen, Denmark

## Abstract

Social interactions among humans create complex networks and – despite a recent increase of online communication – the interactions mediated through physical proximity remain a fundamental way for people to connect. A common way to quantify the nature of the links between individuals is to consider repeated interactions: frequently occurring interactions indicate strong ties, such as friendships, while ties with low weights can indicate random encounters. Here we focus on a different dimension: rather than the strength of links, we study physical distance between individuals when a link is activated. The findings presented here are based on a dataset of proximity events in a population of approximately 500 individuals. To quantify the impact of the physical proximity on the dynamic network, we use a simulated epidemic spreading processes in two distinct networks of physical proximity. We consider the network of *short*-*range* interactions defined as *d* ${\boldsymbol{\lesssim }}$ 1 meter, and the *long*-*range* which includes all interactions *d* ${\boldsymbol{\lesssim }}$ 10 meters. Since these two networks arise from the same set of underlying behavioral data, we are able to quantitatively measure how the specific definition of the proximity network – short-range versus long-range – impacts the resulting network structure as well as spreading dynamics in epidemic simulations. We find that the short-range network – consistent with the literature – is characterized by densely-connected neighborhoods bridged by weak ties. More surprisingly, however, we show that spreading in the long-range network is quite different, mainly shaped by spurious interactions.

## Introduction

Social interactions among humans form complex networks. While these interactions have recently begun to occur via many different channels – email, social networks, texts, and calls^1^ – the interactions mediated through physical proximity remain a fundamental way for people to connect^2^. A common way to quantify the nature of a link is to consider repeated interactions: frequently occurring interactions indicate strong ties, such as friendships, while ties with small weights can indicate random encounters. Here we focus on a different dimension: rather than the strength of links, we study physical distance between individuals when a link is activated. Using epidemics as an example application, we show that changing of our definition of what constitutes a social tie based on the distance of pairs of individuals leads to strong structural differences in the resulting networks and quantify those differences.

The findings presented here are based on a dataset of proximity events in a population of approximately 500 students at the Technical University of Denmark^3^. These students are densely interconnected via networks of interactions, both virtual (Facebook, calls, texts) and based on physical proximity (both within university campus and outside). The full dataset – known as the Copenhagen Networks Study – contains two years of high-resolution records of students’ activity (the aforementioned networks along with GPS location and questionnaires), collected primarily through smartphones distributed to students at the beginning of their university education. Here, we explore the dynamic network where every person is represented by a node, and two nodes are connected if they are within certain physical distance *d* of each other. While this network is small from the perspective of population-level epidemiological studies, the access to physical proximity sampled at the 5-minute level, provides very a detailed view of possible empirical spreading paths (see Table 1 for details).

**Table 1:** Overview of networks statistics.

	Long-range	Sampled Long-range	Short-range
Number of interactions	1 472 094	269 094	269 094
Number of links	42 838	26 511 ± 68	13 474
Avg. link weight	34.36	10.15	19.97
Number of nodes	464	464	464

The number of interactions is, by definition, the same in the short-range and sampled long-range networks. Those interactions, however, end up distributed on different number of links, resulting in a slight variation in the average link weight. Similarly, the number of links differs slightly across different realizations of the sampled long-range network, here shown as ±standard deviation. The number of nodes (students) is the same in all three networks.

To quantify the impact of the physical proximity on the dynamic network, we use a simulated epidemic spreading processes in two distinct networks of physical proximity. We consider the network of *short*-*range* interactions defined as $d\lesssim 1$ meter, and the *long*-*range network* which includes all interactions $d\lesssim 10$ meters^4^. Below we show that the short-range and long-range networks are fundamentally different in terms of structure and dynamics.

The key novelty of this work arises from the fact that we are able to explore dynamics of two distinct types of spreading mechanisms (in many ways similar to e.g. droplet vs. airborne spreading mechanisms) based on the same underlying empirical behavioral data. Because we are able to consider two fundamentally distinct networks arising from a single underlying dataset, we can be certain that the differences in infection patterns are related solely to differences in how the disease is able to spread on each of the networks. This implies that differences in spreading patterns are not due to other differences in behavior that one might encounter when comparing two disparate datasets of actual human behavior, such as mobility, culture, population density, demographics, etc. Similarly, having both short- and long-range networks directly observable allows us to sidestep creation of synthetic networks via randomization schemes.

In the literature on physical proximity, the tacit rules of human interactions in physical space have been an object of interest since the 1950’s^5–7^. Yet little is known about how the structure of person-to-person proximity networks change as we vary the definition of which distance between two individuals corresponds to a connection between the two. Previous research into proximity networks has been based on self-reported data^6,8,9^ or tightly-controlled laboratory observation^5^.

We expect the social network of individuals to be closely related to the structure of the short-range network, but with some differences. This similarity arises because, in social networks, the difference between friend and stranger is typically expressed via different personal spaces for each social category^6^. Interactions with individuals with whom we are not familiar tend to occur at larger distances (we use term ‘interaction’ for all proximity events, including the long-range network). Since people function in bounded spaces, however, we do not have complete freedom to only allow friends to be physically close to us. Rides on buses, random meetings in elevators, or busy dining halls force us to be in close proximity to strangers. Thus, while the majority of our proximity interactions are with friends and families, our interactions network is not fully explained by the underlying social network, as expressed by, for example, link strengths. The long-range networks contains all of the links in the short-range network, but in addition also spurious connections to people passing by and the ‘familiar strangers’^10,11^, those individuals we encounter repeatedly but have never gotten to know. Thus, considering the proximity of pairs engaging in interactions and moving beyond simply considering the weight of the links in the network, provides a new source of information regarding potential spreading paths.

From the network science literature we know that social networks exhibit non-trivial structure on every level from degree distribution^12,13^, over motifs^14,15^, to communities^16–18^, and at time even an overall hierarchical organization^19^. In the light on the research on physical proximity discussed above it is interesting to keep these key findings from the social networks literature in mind as we explore the differences between the short-range and long-range networks.

## Results

The proximity networks are based on Bluetooth scans providing a measure of pairwise proximity between *N* = 464 highly-connected participants – freshmen students at a large university^3^. We define an *interaction* between users *i*, *j* in a 5-minute timebin *t* (the smartphone were configured to scan for nearby devices every 5 minutes) as *γ*_*ijt*_ = *s*, where the signal strength *s* is reported by the handsets as received signal strength indicator (RSSI). Two users are considered to be interacting within a given timebin if their phones registered each other at least once in that timebin, regardless of the reported signal strength. This densely-connected dynamic network of all Bluetooth interactions is based on a total of 1472 094 interactions, taking place over 28 days. RSSI, measured in *dBm*, is defined as the observed signal power relative to 1 *mW*.

### The long-range, sampled long-range, and short-range network

The *long*-*range* network is created by interactions occurring at any distance covered by Bluetooth range, between 0 and 10–15 meters. In order to capture only close range interactions, we establish the *short*-*range* network by selecting the subset of interactions with *γ*_*ijt*_ ≥ −75 *dBm* corresponding to distances of approximately 1 meter or less^4^ (see Supplementary Information for additional details on the choice of threshod). The short-range network consists of *f* = 18.3% of all interactions.

Since the short-range network contains only a fraction of all interactions, the simulated spreading processes taking place on this network are trivially slower and smaller than processes occuring on the long-range network. The intuitive reason for this is that with an average of one fifth of the interactions, a node in the short-range network has correspondingly fewer opportunities of spreading a disease than in the long-range network. The difference in number of interactions therefore prevents us from directly comparing the interplay between structure and dynamics of spreading processes for the short-range and long-range networks using simulated disease models with the same parameters.

**Figure 1:**
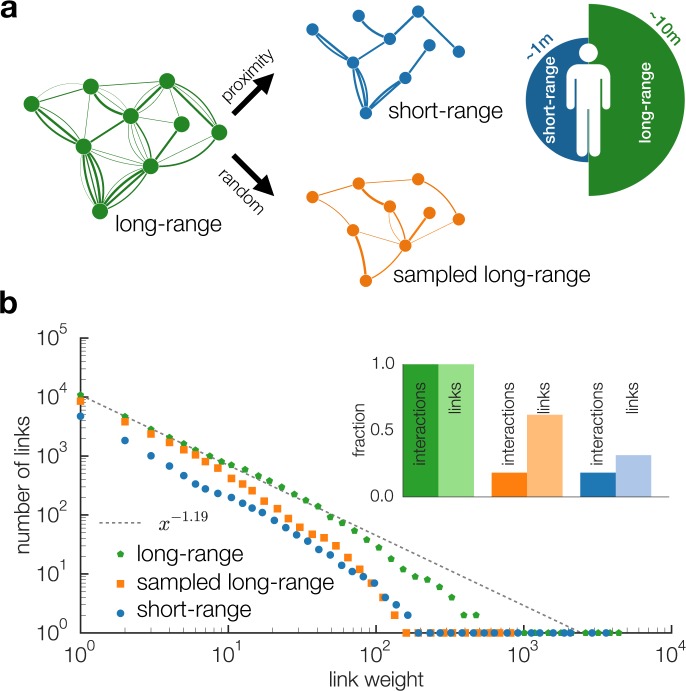
The network of close proximity interactions. (**a**) The full network contains interactions with signal strength used as a proxy for a proximity. In this illustration the dynamic network is integrated over time; edges represent single interactions between participants, and line-width indicates physical proximity. From this full network, corresponding to all edges that support full-range transmission, we create the short-range network by only considering interactions with *γ*_*ijt*_ ≥ −75 *dBm*. The sampled long-range network contains the same number of interactions but chosen at random. (**b**) The link weights (*i*, *j*) are broadly distributed. The dashed line is a power-law *p*(*x*) ~ *x*^−*α*^ with *α* = 1.19 inserted as a guide to the eye. The sampled long-range network (orange) has the same number of interactions *γ*_*ijt*_ as the short-range network (blue), but maintains 62% of links, compared to only 31% of links remaining in the short-range network (inset).

In order be able to compare directly, we create a *sampled long*-*range network*, which contains the same fraction of interactions as the short-range network, but chosen at random among all interactions (see Fig. 1a). As we argue below, the sampled long-range network thus contains both close and distant interactions and shares most topological properties with the full long-range network, while based on precisely the same number of interactions as the short-range network.

### Link weights in the three networks

We start our analysis by studying similarities and differences in the the distribution of link weights between the three networks (long-range, sampled long-range, and short-range). For each of the networks, we calculate the weights as described below, using the long-range network as an example. We first create an adjacency matrix *A*_*i*×*j*×*t*_ with timebins *t* containing interactions aggregated over 5 minute intervals corresponding to the Bluetooth scanning rate. This matrix has entries *a*_*ijt*_ = 1 when an interaction is present and *a*_*ijt*_ = 0 otherwise. The weight *w*_*ij*_ of a link connecting two individuals is defined as the total number of interactions occurring on that link ${w}_{ij}={\sum }_{t}\,{a}_{ijt}$. Note that because the sampled long-range network is generated by sampling interactions at random from the full network, it is possible to calculate the weight distribution for this network analytically.

We use a number of closely related (but distinct) terms to describe connections between pairs of individuals. A quick overview of terms are: *Interaction*: A single measurement of proximity between a pair of individuals. *Signal strength*: The RSSI measured by a smartphone for a single interaction. The signal strength can be considered a measure of distance. *Link*: An abstract description of the connection between two individuals, and implies at least one interaction. Links are sometimes denoted *ties* or *connections* in the literature. *Weight*: Number of interactions observed on a given link; sometimes called *strength* in the literature.

As shown in Fig. 1b, the distribution of link-weights in all three networks is broad with many weak links (containing few interactions) and a small number of links of very high weight. The short-range network and the sampled long-range network contain the same number of interactions, but the number of resulting links in the two networks is strikingly different. The approximately 1.4 million interactions in the full long-range network are distributed across 42 838 links, resulting in an average link-weight of a little over 34 interactions per link. We create the short-range and sampled long-range networks by removing 81.7% of the interactions from the full long-range network, leaving 269 094 interactions in both of these networks. The resulting number of links is much higher in the long-range network. Averaged over 100 realizations, this network has 26 511 ± 68 links, corresponding to around 61.9% of the links in the full long-range network. In contrast, the short-range network has only 13 474 links corresponding to only 31.5% of the links in the full long-range network. These differences are illustrated in the Fig. 1b inset.

Let us investigate these difference with respect to link weight in further detail. First, let us consider the weakest links. In terms of low weight links the sampled long-range network simply retains around *f* = 18.3% of the long-range network’s links, with small differences. The reason for these differences can be understood by considering links with weight 1. Of course, (100–18.3)% of links with weight 1 are removed, but the sampling process also creates new links of weight 1 by down-sampling the weight of some number of links with weight 2, 3, etc. In the short-range network a much higher fraction of links with weight 1 are removed, this network has about half as many links with weight 1 as we find in the sampled short-range network.

Now, considering high-weight links we find that these links in the short-range network are relatively unaffected by removing interactions according to physical distance: in the short-range network we find that the highest-weight links typically maintain ~80% of their interactions). This is in stark contrast to the sampled long-range network, where link-weight is depleted in proportion to the sampling fraction, and high-weight links maintain only ~18% of the interactions from the full long-range network.

In summary, the weight distribution in the short-range network suggests that friends (with high-weight links) tend to be physically close and that most low-weight links correspond to random encounters (encounters between strangers), consistent with results on interaction distance from both quantitative measurements^4^ as well as sociology^6^.

### Differences in local structure

The key comparison is between the short-range network and the two long-range networks. Since our sampling is uniform over interactions, we expect the sampled long-range to be structurally very similar to the full long-range network, with weights decreased proportional to the down-sampling fraction. As we discuss above, however, many low-weight links disappear as part of the sampling process, and the overall network structure is complex, reflecting non-trivial and highly correlated underlying social behaviors. Therefore, it is useful to quantitatively confirm that the structure of the long-range and sampled long-range remain remarkably similar – and distinct from the short-range network.

**Figure 2:**
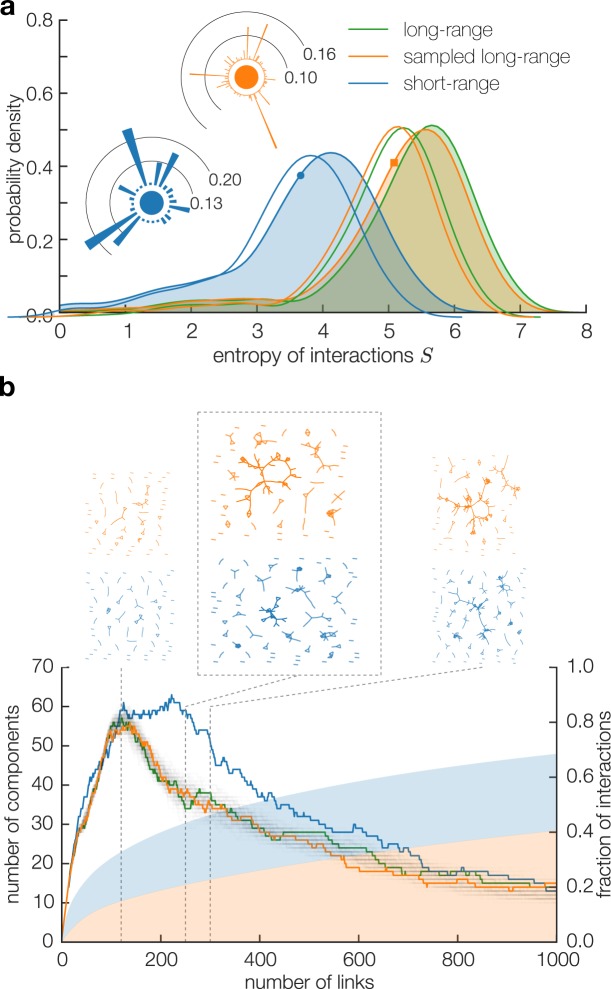
Difference in network structure. (**a**) Entropy of interactions. For every node *i* in the network we calculate node entropy *S*(*i*), see main text for the definition. Entropy values in the sampled long-range network are averaged per user over 100 random realizations of the sampled long-range network. Insets illustrate the link weights for a representative single node (entropy 3.6) in the short-range network (blue) and in the sampled long-range network (orange, entropy 5.1), values indicated by markers on the distributions. Note the similarity between distribution of entropies for the long-range and sampled long-range networks. (**b**) (upper panels) Network snapshots, showing the network structure for the sampled long-range (orange) and short-range (blue) networks at points indicated on the plot below. Note how the short-range network remains separated in small disconnected component longer than the long-range network. (lower panel) The horizontal axis shows changes to network properties as we add links one by one, starting with the strongest link. The green, orange, and blue line-plots show the number of connected components in the network. At around 120 links added, the long-range (green) as well as the sampled long-range network (orange) takes significantly longer to begin to become connected and show a decreasing number of components. Specifically, the short-range network (blue) remains separated into small neighborhoods until we have added approximately 250 of the strongest links. Thus the percolation process starts significantly later in this network. The orange line is an average over 100 random realizations of the sampled long-range network. Each realization is shown as a transparent gray line, illustrating that the network structure is consistent across samples. Also importantly, the fraction of interactions (shaded plots in blue/orange) within each network as we add the strong links also differ strongly between the networks. In the short-range network 250 links correspond to almost 50% of all interactions, whereas the same number of links in the sampled long-range networks contain only around 20% of all interactions.

Starting from the single node perspective, we find important differences between the short-range and the long-range networks. We can quantify this difference using the Shannon entropy. For a node *i*, we start from a link with neighbor *j* with weight *w*_*ij*_ and define $\pi ({w}_{ij})={w}_{ij}/{\sum }_{k}\,{w}_{ik}$ to mean the fraction of the node’s total interactions taking place on that link. Now, we define the *node entropy* as $S(i)=-\,{\sum }_{j}\,\pi ({w}_{ij})\,{\mathrm{log}}_{2}\,\pi ({w}_{ij})$. Since infection probability is approximately proportional to link weight (see SI), this quantity can be interpreted as the expected number of yes/no questions needed to establish which of *i*’s links caused an infection. The distribution of entropy for all three networks is plotted in Fig. 2a. For the short-range network (blue), the distribution peaks at 4 bits, corresponding to an effective group of 2^4^ = 16 potential sources of infection. Comparing the long-range (green) and sampled long-range (orange) networks, we find as expected that the distribution of node entropies are very similar, emphasizing the structural similarity between these two networks. The distribution for the sampled long-range network is created by averaging per-user entropy values over 100 random realizations of the sampled long-range network. Both peak at around 6 bits, corresponding to a larger effective group of 2^6^ = 64 potential sources of infection in this network.

These results provide a striking illustration of how the close proximity zone is preferentially reserved for strong ties (e.g. friends or acquaintances) while the distant zone is a more public space where many more random interactions happen, resulting in a correlation between physical proximity and tie strength as reported in ref. ^9^.

### Meso-level structural differences

In the previous section we showed that in the short-range network a large fraction of interactions takes place on high-weight links. We now study the interplay between meso-level network structure and link-weight in the short-range and long-range networks. Specifically we are interested in the structures formed by the highest weight links. To explore these, we start building the networks from empty, adding their respective strongest links one-by-one. As links are added, we keep track of the number of connected components in the network as well as total weight of interactions added through the links, revealing the differences in the networks with respect to the structures created by the heaviest links.

Figure 2b illustrates how the process of adding links gradually grows the long-range and short-range networks, respectively. In the lower panel of Fig. 2b we show the number of the connected components and total number of interactions in the networks as the links are added. First, notice that the full and sampled long-range networks display identical behavior, with number of neighborhoods peaking with approximately 120 strongest links added. This behavior is consistent across 100 random realization of the sampled network. This is in contrast to the short-range network, where the number of components continues to grow up to 240 heaviest links in the network.

In both types of networks, the strongest links in the network first create small isolated neighborhoods of highly interacting nodes. Figure 2b (upper panel) shows snapshots of the sampled long-range (orange) and short-range (blue) networks at points (120, 250, 300 links) indicated on the plot below, illustrating this point. We see that at 250 strongest links the long-range network, a large connected component is beginning to form, making the network significantly more connected. At this point, the short-range network, however, is still divided into many small neighborhoods. We also note that while the *x*-axis indicates the absolute number of the heaviest links added to the networks, the total number of interactions included in the networks at any number of links is strikingly different. In fact, it is important to underscore just how large a fraction of interaction are concentrated on the high-weight links. The short-range network has a total of 13 474 links and the sampled long-range network has ~26 500 links. Figure 2b (bottom panel), however, shows that in the short-range network the 250 strongest links in the network account for approximately 50% of the interactions. In the long-range network the picture is less skewed. Here, the top 250 links account for approximately 25% of the interactions. Thus, while the percolation transition occurs for a very small number of high-weight links in both networks, these links include a large fraction of the total number of interactions.

Our analysis shows, therefore, that the short-range network not only contains fewer links than the sampled long-range network, but that the configuration of the heaviest links is more fragmented than in the long-range case. This structural property of the short-range network, the highly-connected neighborhoods bridged by weak ties, is consistent with well known structures found in other social networks, such as mobile phone networks and online social networks^17,20–22^. In the long-range network, however, this structure is less pronounced, obscured by the presence of spurious links, distinct communities bridged by a small number of strong links not present in the short-range network.

### Spreading process is captured in neighborhoods

Having investigated differences between short- and long-range networks with respect to structure, we now explore how the differences based on how diseases spread on the networks. Using a simple Susceptible-Infected-Recovered (SIR) model, we run simulations of a disease spreading across the networks. Our model is intentionally simplistic, intended to illustrate the structural differences between short- and full-range transmission, rather than emulate a specific disease. We use the actual temporal sequence of proximity interactions observed in the data, choosing parameter values to create a situation where large outbreaks are likely, but not guaranteed (see Methods for details of the epidemic modeling). While we report results for a specific choice of parameters and a single realization of the sampled long-range network, these results are robust across a wide range values of the transmission parameters and realizations of the sampled network.

**Figure 3:**
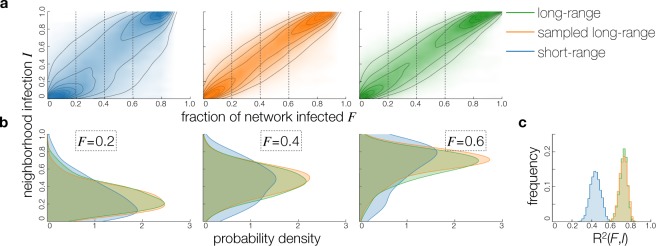
Dynamics of the spreading process. For each infection event, occurring on link *w*_*ij*_, where node *i* infects node *j*, we measure which fraction *I*_*j*_ of the node’s direct (1-hop) neighborhood has already been infected. We define ${I}_{j}={W}_{\{-i\}}^{-1}\,{\sum }_{k\in  {\mathcal I} (j),k\ne i}\,{w}_{jk}$, where ${\mathcal I} (j)$ is the set of *j*’s infected neighbors and ${W}_{\{-i\}}={\sum }_{k\ne i}\,{w}_{jk}$ is the sum of all weights excluding the infecting link. (**a**) Plot of spreading process over 500 simulations. And increasing fraction of nodes are infected (*F*), we observe that nodes with different neighborhood infection levels (*I*) are infected. Kernel density outlines (using Gaussian kernel and silverman bandwidth) illustrating how a broader range of neighborhood infections can be observed in the short-range network (blue). (**b**) Cuts of distribution of *I* at three values of *F* (0.2, 0.4, 0.6, points indicated by vertical lines in the top plots), showing that distribution of neighborhood infections is broader in the short-range (blue) network. (**c**) Distribution of *R*^2^ of a linear model fitting infection of the neighborhoods *I* to the progress of infection (measured as fraction of network infected *F*), calculated for each of the aforementioned 500 realizations of an epidemic. The distribution of *R*^2^ peaks at around 0.4 in the short-range network versus 0.75 in the two long-range networks.

Based on the structural analysis, our hypothesis is that, in the short-range network, the simulated pathogen tends to be more contained within small sets of highly interacting individuals. We quantify the contained-in-communities behavior as follows. For each infection event, occurring on link *w*_*ij*_, where node *i* infects node *j*, we measure which fraction *I*_*j*_ of the node’s direct (1-hop) neighborhood has already been infected. Since this is a weighted network, we define ${I}_{j}={W}_{\{-i\}}^{-1}\,{\sum }_{k\in  {\mathcal I} (j),k\ne i}\,{w}_{jk}$, where ${\mathcal I} (j)$ is the set of *j*’s infected neighbors and ${W}_{\{-i\}}={\sum }_{k\ne i}\,{w}_{jk}$ is the sum of all weights excluding the infecting link. A value of *I*_*j*_ = 0 indicates that no-one in the direct neighborhood besides the infecting node has been yet infected; a value of *I*_*j*_ = 0.5 indicates that neighbors accounting for 50% of link weights connecting to *j* have already been infected. Figure 3a shows a kernel density estimation of *I* as a function of the fraction of infected nodes, based on 500 runs of the spreading process in the short-range (left), sampled long-range (middle), and long-range (right) networks.

In the case of the short-range network, we observe behavior which suggest that the spreading agent is indeed slowed by neighborhoods, consistent with behavior of both simulated and real spreading processes found in the literature^23–27^. As is evident from Fig. 3a, early in the epidemic outbreak, when the fraction of infected nodes is low, the disease agent can saturate small neighborhoods and infect new nodes in neighborhoods, where a large fraction (*I* > 0.80) of neighbors are already infected. Conversely, it is still possible to find neighborhoods with a low fraction (*I* < 0.20) of infected nodes very late in the outbreak. These effects are possible because the spreading agent does not jump easily between neighborhoods of densely connected nodes.

The disease spreading is very different in the full and sampled long-range cases. In contrast to the contained-in-communities picture, the infection progresses smoothly through the network. In the long-range networks, the neighborhood infection is more closely proportional to the fraction *F* of the total network infected. Cuts at particular levels of overall network infection *F* in Fig. 3b show that the pattern of more spread-out *I* in the short-range network is consistent through the spreading progression and across random starting conditions (seed node and time) Visually, the distributions of *I* at given *F* are narrower for the long-range networks, with peak values of neighborhood infection *I* closer to values of overall network infection *F*. To quantify this effect, we consider the distribution of *R*^2^ of a linear model fitting infection of the neighborhoods *I* to the progress of the infection (fraction of network infected *F*), calculated for each of the aforementioned 500 realizations of an epidemic, the distribution of *R*^2^ peaks at around 0.4 in the short-range network vs 0.75 in the two long-range networks, as shown in Fig. 3c. This indicates that direct proportionality between the global (*F*) and local (*I*) infection level is a significantly better model for the long-range networks.

Thus we find, that while – in the short-range network – the infection tends be captured inside closely connected communities, the picture is quite different in the long-range network. While both types of behavior has been described in the literature^8,23–28^, the important finding in this context is that the two networks are representations of *the same underlying behavioral data* originating from a single population. These findings underscore how long-range spreading dramatically taps into spurious connections outside the social networks, resulting in fundamentally different types of spreading – in some ways mimicking the differences between droplet and airborne spreading mechanisms^29–32^.

### Community structure increases infected-infected interactions

**Figure 4:**
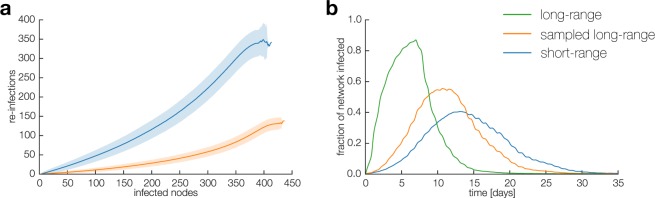
Dynamics of the spreading process. Results for 10 000 SIR simulations. (**a**) As the infection progresses through the network, we keep track of how often the a link between two infected nodes is activated. Shaded areas indicate one standard deviation. (**b**) The overall result is significantly slower outbreaks in the short-range network than in the long-range networks.

Our analysis of link weights showed that the short-range network tends to have fewer links with more interactions on each link. But why is the disease trapped within communities in the first place? One of the reasons that an infection remains ‘stuck’ in a neighborhood is that a disease can only spread via interactions between infected and susceptible nodes. Thus, if a local group is fully infected, we tend to see a large fraction of infected-infected interactions, which cannot help spread the disease. In Fig. 4a we quantify this tendency, by plotting how frequently infected-infected are active in the sampled long-range and short-range network, respectively.

We observe a clear difference between two networks. In the sampled long-range network, where the local connection patterns have high entropy, there is only a low level of activity among infected or recovered individuals. The spreading agent quickly reaches the entire network due to a large number of available susceptible-infected links. This behavior is in contrast to the short-range network, where infected-infected interactions present a larger fraction of interaction events. Thus, as above, given the same number of interactions and the same underlying behavioral data, outbreaks are significantly slower and more contained in the short-range network relative to the sampled long-range case (Fig. 4b).

### Statistics of spreading outcomes

**Figure 5:**
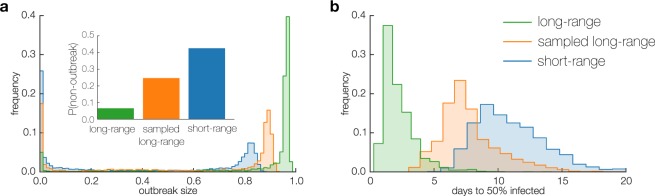
Statistics of spreading. (**a**) In the short-range network the outbreaks are smaller than in the sampled long-range network, even though these two contain exactly the same number of interactions. The probability of outbreak being contained – reaching only a small fraction of the network – is also higher in the short-range network (inset). (**b**) When outbreaks happen, the time to 50% of the network becoming infected is significantly longer in the short-range network, because the spreading is captured within small neighborhoods.

Finally, in Fig. 5 we summarize a number of statistics related to disease spreading in the three networks. These results confirm that the structural differences between the short-range and long-range interaction networks discussed above lead to reliably different outcomes in simulated epidemics. Firstly, in Fig. 5a, we show that when the outbreaks do happen in the short-range network, they are smaller in terms of total number of nodes infected. Moreover, the probability that an outbreak is contained – reaching only a small fraction of the network (<20%) – is higher in the short-range network than in the long-range networks (Fig. 5a inset). Finally, the time an infection needs to reach 50% of the short-range network is significantly longer, with the peak of the distribution for sampled long-range network occurring after 7 days, while the short-range network the peak is delayed to 10 days (Fig. 5).

Thus, consistent with the literature short-range short-range interactions are organized in a way that slows down spreading relative to the long-range case. The sampled long-range network features precisely the same number of interactions as the short-range network, but is structurally more similar to the full long-range network according to the measures considered here. Our results show that taking the physical distance of interactions into account results in networks that can significantly alter the outcome of a simulated outbreak. The qualitative behavior described above is reproduced across a wide range of parameter values.

## Discussion

We have demonstrated a strong structural difference between the short-range networks that support short-range transmission processes and the long-range networks that support transmission across distances up to 10 meters. Summarizing our findings, we find that the proximity of interactions correlates with link-weight: on average we stay closer to our friends. In the short-range network, we find spreading patterns consistent with our knowledge of spreading on various online social networks and modeling studies^23–27^. In the long-range network we observe a large proportion of proximity interactions between individuals with weak or absent social ties, resulting in a complex local network structure. This non-social ‘noise’ in the network allows for faster and more powerful outbreaks to take place, even when considering the exactly same number of interactions, consistent with results of synthetic proximity-aware spreading simulations^33^.

It is, of course, well known that that the definition of ‘interaction’ impacts the network structure and spreading dynamics. For example, networks of sexual contacts are analyzed separately from other types of pathogen spread^34,35^, even though both types of networks are physical interactions networks. A central work in understanding role of physical proximity is by Read *et al*.^8^, where questionnaire data regarding ‘close’ and ‘distant’ interactions were collected from 49 participants over 14 non-consecutive days. This study, however, did not address how differences in mode of transmission can affect the network of infections. Recently, a multitude of new approaches have been developed for collecting data regarding close interactions with the purpose of modeling spreading using various methods, including Bluetooth, RFID, and questionnaires^8,28,36–39^.

Here we argue that from the perspective of a spreading agent, the relatively subtle difference of what ‘interaction’ is in the short-range and long-range networks makes an important difference, even given the same underlying social system. Our results suggest that long-range spreading is less related to the underlying social network and closer to a well-mixed system than simulations on purely social structures might lead one to suggest.

## Methods

### The dataset

The dataset used in this paper comes from the Copenhagen Networks Study^3^. We use one month of data (February 2014). Out of 696 freshmen student participants active in that month we chose students with at least 60% of Bluetooth observations present (resulting median 80%) and who belong to a single connected component. Observations are defined as 5-minute bins in which the user has performed scans, whether the scans contained any devices or not. Since Bluetooth scans do not result in false positives, we symmetrized the observation matrix (resulting in an undirected network), assuming that ${\gamma }_{ijt}\iff {\gamma }_{jit}$. This results in improved data coverage, with a median of 85% of 5-minute containing data. More information regarding the dataset is provided in the Supplementary Information.

### RSSI and interaction distance

The received Signal Strength Indicator (RSSI) can be used to estimate the distance between wireless devices^40^. Sekara & Lehmann^4^ showed the stability of RSSI in modern mobile phones; the same phones were used in the Copenhagen Networks Study. Based on these results, we use *γ*_*ijt*_ = *RSSI* ≥ −75 *dBm* as an indicator that an interaction was closer than 1 meter. This value can be considered a conservative estimation, as the measurements in ref.^4^ have been performed without obstacles. Thus, we expect that *γ*_*ijt*_ ≥ −75 *dBm* may not include all the close interactions, but it should not include distant interactions. When the interaction matrix is symmetrized, we take the smallest distance (largest RSSI) that happened between users in given timebin *γ*_*ijt*_ = *γ*_*jit*_ = min(*γ*_*ijt*_,*γ*_*jit*_).

We note that the approach presented here has some limitations. While all mobile phones used for data collection in the study were the same model and the obtained RSSI values are comparable in this sense, it is important to emphasize that our distance threshold is noisy; RSSI may differ depending on where the phone is placed, environmental conditions, etc. In that sense, our results can be considered a *lower bound* of the difference between the two types of networks, since a perfectly noisy threshold would produce two randomly sampled networks with no difference between them.

### Epidemic simulations

To show the dynamics of the spreading process in the droplet and airborne networks we use a simple Susceptible-Infected-Recovered (SIR) simulation. We run a large number of simulations (*N* = 10 000) on the full temporal network, where every interaction between Infected and Susceptible participants can lead to infection with probability *β* = 0.02. Users stay in Infected state for *μ*_*t*_ = 7 days, after which they are moved to Recovered state and cannot be re-infected. The starting time bin and seed node are chosen at random in every simulation and used for simulation on all three networks (long-range, sampled long-range, and short-range). We use one month of data (28 days, 8 064 5-minute timebins) with periodic boundary conditions, having the 28 days repeating indefinitely. The parameter values are chosen so that outbreaks are likely, but not guaranteed and with sizes that do not trivially saturate the entire network. The parameters themselves as well as resulting epidemic curves (with peaks between 7 and 14 days) are consistent with these reported in the literature regarding both simulated and observed flu outbreaks^8,37,41^. This model is intentionally simplistic, intended to illustrate the structural differences between full- and short-range transmission, rather than emulate a specific disease. The qualitative behavior of our analysis is unchanged across a wide range of parameter values.

## Electronic supplementary material

Supplementary Information
