# Supplementary material for: How Physical Proximity Shapes Complex Social Networks
Source: Sci Rep. 2018 Dec 7;8:17722. doi: 10.1038/s41598-018-36116-6 (PMC6286340; doi:10.1038/s41598-018-36116-6)
Supplement: Supplementary file 1 — Supplementary Information [file 41598_2018_36116_MOESM1_ESM.pdf]

Supplementary Information for How Physical Proximity Shapes Complex Social Networks

Arkadiusz Stopczynski[1,2], **Alex 'Sandy' Pentland**[2], **and Sune Lehmann**[1,3,*]

[1]Technical University of Denmark, Lyngby, Denmark
[2]Media Lab, Massachusetts Institute of Technology, Cambridge, MA, USA
[3]Niels Bohr Institute, University of Copenhagen, Copenhagen, Denmark
[*]sljo@dtu.dk

1 The dataset

For every participant in the Copenhagen Networks Study[1] in February 2014 (696 active users) we calculate the number of 5-minute timebins in which we have data on Bluetooth obsevations (whether they contain any other participant in the study or not). Out of this population we choose users with data in at least 60% of timebins covered (Figure 1). As the Bluetooth scans do not yield false positives (devices that are not actually present during the scan), we make the discovery network symmetric by assuming that if user i observed user j in timebin t the opposite is also true. After the matrix is made symmetric, the median time coverage for the users reaches 84%. The degree and node strength have broad distribution in the full network (Figure 2). The strength of the nodes has a slightly longer tail in the intimate network, indicating existence of a few nodes with very strong links that are reduced in the random sampling.

The temporal dynamics of the social networks show an expected daily and weekly pattern as quantified by the number of active links in the network in 1-hour timebins (Figure 3). Weekdays display morning and afternoon peaks of activity, with decreased activity during lunchtime. Fridays show a significantly reduced afternoon peak but a much more pronounced evening peak, indicating social gatherings. Most of the weekends show significantly smaller activity, except for occasional parties (for example on Saturday evening in day 22). This shows that although the analyzed network is primarily driven by work-related interactions, it also contains a non-trivial number of social interactions.

The difference in the entropy of interactions between intimate and (sampled) ambient network (shown in the main text) is not explained by a simple change in the degree of the nodes. Plotting the difference in entropy vs. change in degree reveals only week correlation $R^2 = 0.17$ as shown in Figure 4.

1.1 Link weights and spreading

Strong links in the networks also tend to be more infectious. This is shown in Figure 5. The correlation is strongest in the sampled ambient network ($R^2 = 0.90$). In the intimate (and ambient) network the infectious power of the strongest links is lower than we would expect from a linear model, due to the high clustering of these links in the tightly-connected neighborhoods (as shown in the main text).

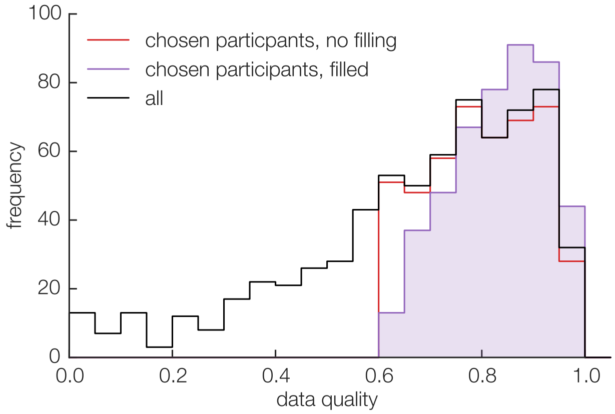

Figure 1. Quality of the data. We calculate in how many 5-minute bins we have Bluetooth data for the users. Out of all users we chose these with data quality of over 60%. After filling the observations by making the observations matrix symmetric, the data quality still improves slightly (shaded area).

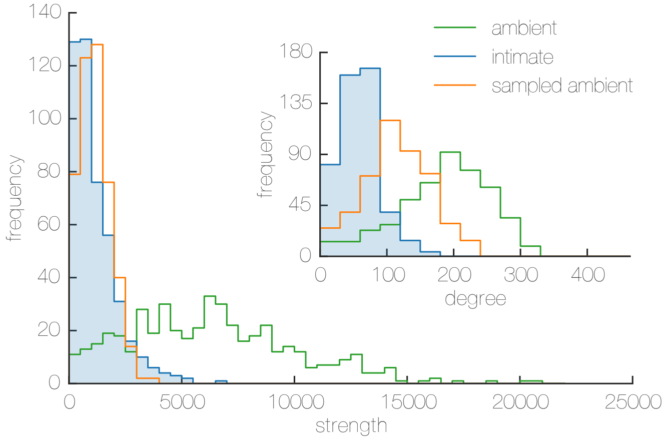

Figure 2. Degree and strength in the network.

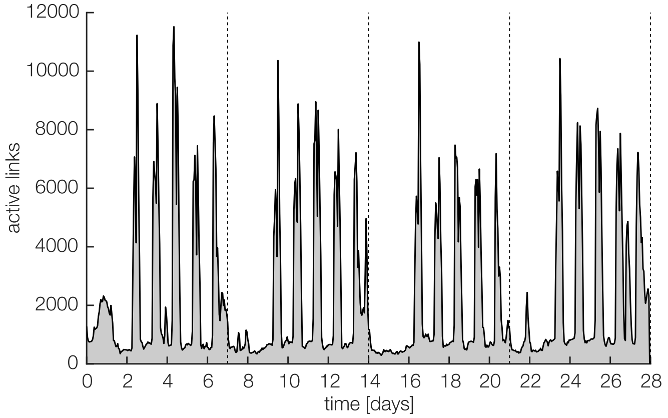

Figure 3. Temporal dynamics in the network. Number of active links in 1-hour temporal bins. Dotted lines show Friday-Saturday midnight.

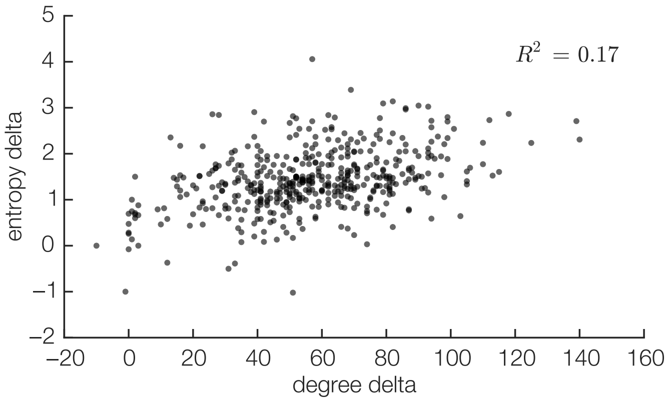

Figure 4. Correlation between difference in degree and entropy between intimate and sampled ambient networks. The change in the degree does not explain the change in the entropy.

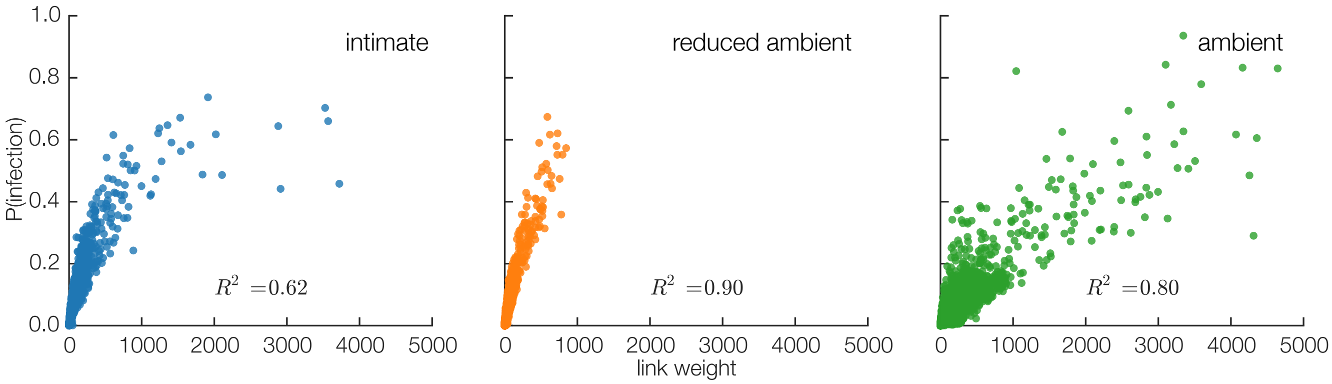

Figure 5. Correlation between link weight and probability of infection happening on this link (in any direction).

2 RSSI and interaction proximity

RSSI is a noisy proxy for distance. Numerous lab experiments have shown strong (logarithmic) relation between RSSI and distance[2–4]. Recently, the relation between proximity measured by RSSI and social ties has also been shown[5]. The distribution of RSSI observations decays exponentially (Figure 6). The dataset used here does not contain data allowing for direct validation of RSSI as proximity indicator, as it does not include any signal that can reliably identify proximity with higher or even comparable resolution. Instead, we use the overlap of other sensed Bluetooth devices to show how RSSI relates to physical proximity.

For every observations between participants we calculate the jaccard overlap between all the other devices they sensed in the timebin. For example, for a 10 minute timebin—which on average contains two scans—we take the value of RSSI as the smallest RSSI between the users in that timebin and for every user we create a set of all unique devices sensed in that period (usually in two scans). Distant interactions (with small RSSI) show smaller overlap of the discovered devices (Figure 7). This is true for timebins (over which the overlap is calculated) spanning over four orders of magnitude, including extremely short windows of 5 seconds and long windows of 4 hours. In the timebins shorter than used Bluetooth scanning interval (5 minutes) the overlap between single scans is calculated (and the scans can be separated in time up to the size of the timebin). For the longer timebins, overlap between devices sensed in multiple scans is used. For the high RSSI values there is a negligible fraction of observations that do not show at least 50% overlap (distributions in Figure 7), which confirms that higher RSSI values are indicative of closer physical proximity.

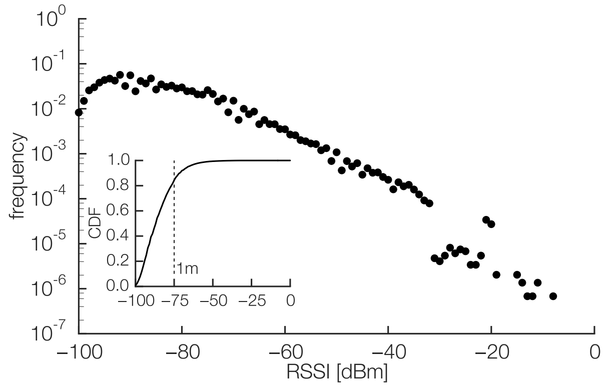

Figure 6. Distribution of RSSI for the full network. Short-range interactions (1m and closer) account for 18.3% of all interactions (inset).

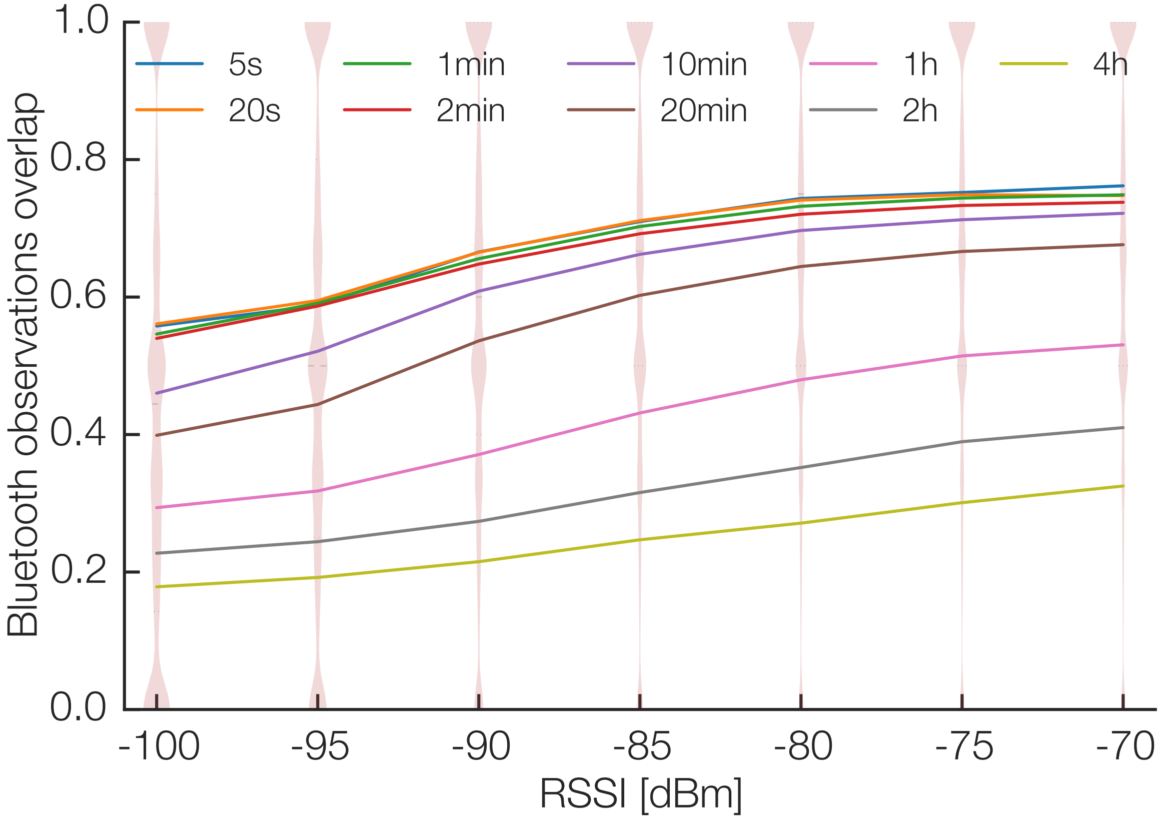

Figure 7. Jaccard overlap between sensed Bluetooth devices depending on the RSSI between participants. Higher RSSI indicates closer physical proximity which also results in the higher overlap. Observation are binned in $5dBm$ bins. Plots for different time windows, across which the overlap is calculated. For all the time windows the higher RSSI indicates higher overlap of discovered devices. Lines indicate mean values. Distributions are shown for $t = 10$ min).

References

1. Stopczynski, A. *et al.* Measuring large-scale social networks with high resolution. *PLoS ONE* **9**, e95978 (2014). DOI 10.1371/journal.pone.0095978.

2. Aamodt, K. Cc2431 location engine. *Appl. Note AN042 (Rev. 1.0), SWRA095, Tex. Instruments* (2006).

3. Feldmann, S., Kyamakya, K., Zapater, A. & Lue, Z. An indoor bluetooth-based positioning system: Concept, implementation and experimental evaluation. In *International Conference on Wireless Networks*, 109–113 (2003).

4. Kotanen, A., Hannikainen, M., Leppakoski, H. & Hamalainen, T. D. Experiments on local positioning with bluetooth. In *Information Technology: Coding and Computing [Computers and Communications], 2003. Proceedings. ITCC 2003. International Conference on*, 297–303 (IEEE, 2003).

5. Sekara, V. & Lehmann, S. The strength of friendship ties in proximity sensor data. *PloS one* **9**, e100915 (2014).
